# Immunological and tissue reactions to titanium particles generated by the mechanical decontamination of dental implants: In vitro and in vivo study

**DOI:** 10.4317/medoral.27171

**Published:** 2025-10-17

**Authors:** Javier Gil, Darcio Fonseca, Manuel Fernández-Domínguez, Pedro Fernández-Domínguez, Sayuri Akagi-Camacho, Jorge Toledano-Serrabona, Erika Vegas-Bustamante, Octavi Camps-Font, M Ángeles Sánchez-Garcés

**Affiliations:** 1 Bionspired Oral Biomaterials Interfaces. Department of Materials Science and Engineering, Barcelona East School of Engineering, Universitat Politècnica de Catalunya Av. Eduard Maristany 16, 08019 Barcelona. Spain.; 2 Department of Translational Medicine CEU San Pablo University. Urbanización Montepríncipe, 28925 Alcorcón Madrid, Spain.; 3 Faculty of Dentistry, Camilo José Cela University. C/ Castillo de Alarcón, 49 Urb. Villafranca del Castillo. 28691 Villanueva de la Cañada. Madrid.; 4 Bellvitge Biomedical Research Institute (IDIBELL), Department of Oral Surgery and Implantology, Faculty of Medicine and Health Sciences, University of Barcelona, 08907 Barcelona, Spain.; 5 Faculty of Dentistry, Universidad Alfonso X El Sabio, C. de Emilio Muñoz, 13, 28691 Madrid, Spain.; 6 Department of Dental Research, Federico Henriquez y Carvajal University, Santo Domingo 10106, Dominican Republic

## Abstract

**Background:**

Mechanical decontamination of biofilm, or implantoplasty, is a commonly employed technique for managing peri-implantitis. However, the inflammatory response and in vivo behavior of tita-nium (Ti) particles released during this procedure remain underexplored. This study aimed to evaluate the cytotoxic, inflammatory, and osteogenic effects of Ti particles released during im-plantoplasty, as well as their in vivo behavior

**Material and Methods:**

Titanium particles were generated by following a standardized protocol using drills on 150 commercially pure Ti implants. Cytotoxicity thresholds were determined using THP-1 macrophages and bone marrow-derived mesenchymal stem cells (BM-MSCs). These cells were subsequently cultured with Ti particle-conditioned medium, and inflammatory responses were analyzed using RT-qPCR for markers such as CCR7, TNF-?, IL-1? (pro-inflammatory), and CD206, TGF-?, IL-10 (anti-inflammatory). Cytokine levels were quantified using ELISA. Osteogenic responses in BM-MSCs were assessed by analyzing Runx2, alkaline phosphatase (ALP), and osteocalcin (OC) expression, and ALP activity was measured colorimetrically. In vivo, Ti particles were introduced into mandibular defects in 30 Wistar rats, with histological analysis performed 20 days post-implantation

**Results:**

Ti particles elicited a pro-inflammatory response in macrophages, with increased expression of TNF-? and reduced expression of TGF-? and CD206. Cytokine analysis confirmed elevated IL-1? and reduced IL-10 levels. No significant changes in ALP activity were observed.

**Conclusions:**

Titanium particles released during implantoplasty induce pro-inflammatory responses.

## Introduction

Peri-implantitis represents a significant challenge in implant dentistry, with an estimated 24% of implants requiring revision within 10 years due to bacterial colonization and tissue damage (1). While novel bactericidal implant materials, such as silver nanoparticles, nanotextures, and plasma-applied coatings like polyethylene glycol, show promise, these approaches lack sufficient validation to meet international accreditation standards for medical applications (2) Consequently, implantoplasty (IP) has become a widely adopted technique for managing peri-implantitis due to its practicality and cost-effectiveness.

IP involves mechanically altering the titanium (Ti) surface colonized by biofilm using dental burs, allowing treatment without implant removal. However, the technique has notable limitations. Ti particles released during IP have been associated with inflammatory and cytotoxic effects, as demonstrated in studies by Toledano-Serrabona et al. (3). Furthermore, several authors highlighted the variability in outcomes due to the lack of standardized protocols for instrumentation and post-IP chemical treatments, which can affect surface properties and promote bacterial recolonization (4 , 5).

Ti particles released during IP range from 35 to 50 micrometers in size (4), with some studies indicating that nanometric particles evade aspiration systems (6). Chemical treatments such as hypochlorous acid, hydrogen peroxide, and ozone, while effective in decontaminating surfaces, have been shown to cause hydrogen embrittlement and reduce the mechanical integrity of Ti, further complicating standardization efforts (7 - 9).

The biological implications of these particles extend to inflammatory responses and alterations in tissue integration. For example, Callejas et al. (6) demonstrated toxic effects of Ti particles in vitro, while Kotsakis et al.(5) discussed how inflammatory environments alter Ti oxide stoichiometry, leading to oxygen depletion and potential adverse tissue responses (6 , 8). These findings underscore the need for comprehensive studies evaluating both the immunological and biological effects of Ti particles.

Therefore, the present study aimed to investigate the immunological response to Ti particles generated during IP, characterize their size, morphology, and biological effects, and assess their impact on macrophages and osteogenic differentiation. Additionally, the biological response to these particles was evaluated in vivo using a Wistar rat model to provide insights into their effects on both bone and soft tissue.

## Material and Methods

Titanium metal particles and characterization

Titanium particles were generated by implantoplasty of 150 commercially pure (c.p.) grade 3 Ti implants (Klockner Dental Implants, Escaldes-Engordany, Andorra).

Implantoplasty was performed by a single operator (EVB), following a simplified three-bur protocol described by Costa-Berenguer et al. (10). The procedure was carried out using a GENTLEsilence LUX 8000B turbine (KaVo Dental GmbH, Biberach an der Riß, Germany) under constant irrigation. The surface was sequentially modified using a fine-grained tungsten carbide bur (ISO 514) (reference H379.314.014, KOMET GmbH &amp; Co. KG, Lemgo, Germany), followed by a coarse-grained silicone polisher (reference 9608.314.030, KOMET GmbH &amp; Co. KG, Lemgo, Germany), and finally a fine-grained silicone polisher (reference 9618.314.030, KOMET GmbH &amp; Co. KG, Lemgo, Germany). A new set of burs and polishers was used for each implant. The samples were lyophilized to remove water from the metallic debris released during IP.

Additionally, a commercially available Ti powder (NanoShel®, UK) with a particle size distribution of 30 to 70 nm was used as a control for inflammatory and osteogenic assays.

The specific surface area of the particles was measured using the Brunauer-Emmett-Teller (BET) theory under controlled vacuum conditions with nitrogen as the adsorbate. Particle size distribution was analyzed using a Mastersizer 3000 (Malvern Panalytical®, UK), laser diffraction system, conducted in a wet medium with ethanol as the dispersing liquid. Mechanical and ultrasonic agitation were applied to prevent particle agglomeration. Morphology and chemical composition were examined using scanning electron microscopy (SEM) equipped with energy-dispersive X-ray spectroscopy (EDS), (Neon 40 Surface, Zeiss, Oberkochen, Alemania).

Sterilization of samples of Ti and collection of extracts.

Both IP debris and c.p. Ti powder samples were sterilized with 96% ethanol. Ethanol was removed through three centrifugation cycles at 7200 rpm, followed by washing with Dulbecco's Phosphate Buffered Saline (DPBS). The samples were then incubated in cell culture medium at a concentration of 0.2 g/mL for 72 hours at 37°C to prepare extracts. These extracts were used for all subsequent cell assays, ensuring compliance with ISO 10993-5 guidelines.

Cell culture

The protocols for culturing THP-1 macrophages and BM-MSCs followed the published elsewhere (11). THP-1 macrophages were maintained in RPMI 1640 medium, enriched with fetal bovine serum (FBS) and penicillin-streptomycin, and incubated under controlled conditions at 37°C with 5% CO2. To promote cell adhesion, THP-1 cells were treated with phorbol 12-myristate 13-acetate (PMA) for 6 hours before exposure to titanium particle extracts. Lipopolysaccharide (LPS) was used as a positive control to induce inflammation, while untreated cells cultured on standard tissue culture plastic (TCP) were used as a negative control.

The biological effects of the titanium particle extracts on macrophages were assessed using several assays. Cytotoxicity was evaluated via resazurin reduction tests, which measured cell viability at 24 and 48 hours post-exposure to undiluted Ti extracts and various dilutions (1:2, 1:10, 1:100, 1:1000). Cytotoxicity was evaluated using resazurin reduction assays, which measured cell viability at 24 and 48 hours after exposure to undiluted Ti extracts and various dilutions (1:2, 1:10, 1:100, 1:1000).

The inflammatory response was analyzed by quantifying the expression of pro-inflammatory markers (CCR7, TNF-, IL-1) and anti-inflammatory markers (CD206, TGF-, IL-10) using real-time quantitative polymerase chain reaction (RT-qPCR). Additionally, the release of cytokines, including TNF-, IL-1, and IL-10, was measured in culture supernatants collected at 24 and 48 hours using enzyme-linked immunosorbent assays (ELISA). Three groups were compared: TCP as the negative control, LPS as the positive control, and Ti extracts (Ti).

Bone marrow-derived mesenchymal stem cells (BM-MSCs) were cultured in osteogenic medium supplemented with -glycerophosphate, ascorbic acid, and dexamethasone. To assess the cytotoxic and osteogenic effects of titanium particle extracts, cells were exposed to undiluted Ti extracts and various dilutions (1:2, 1:10, 1:100, 1:1000) and various assays were performed. TCP was used as a control. Cytotoxicity was determined using resazurin reduction assays at days 3 and 7, while RT-qPCR was employed to evaluate the expression of osteogenic markers such as Runx2, alkaline phosphatase (ALP), and osteocalcin (OC) at days 3, 7, 14, and 21. They compared the Ti extracts (Ti) groups versus the osteogenic medium (Osteo) group. Early osteogenic differentiation was further analyzed by measuring ALP activity through colorimetric assays.

In vivo test.

Animal housing and surgical procedures were conducted at the Experimental Medicine and Surgery Service (SMCEXP) of the General Hospital of Defense Gómez Ulla under ethical approval (Reference 01/2022). Twenty adult Wistar rats (50% male, 50% female), with weights ranging from 210 to 330 g, were used, yielding 40 study alveoli (20 experimental with metal particles and 20 controls).

Surgical procedures were performed under natural and artificial lighting at a con-trolled temperature of 20-24°C. Food and water were provided ad libitum, and cage cleaning followed SMCEXP's standard operating procedures. All animal handling complied with RD 1201/2005, ensuring humane treatment.

The rats were anesthetized with intramuscular ketamine and medetomidine (75 mg/kg). Once anesthetized, they were placed in lateral decubitus on a cork surgical board, with their mouths immobilized open using sutures fixed to the cork. Alternate decubitus positions allowed for the extraction of two mandibular molars (one left, one right). Sterile gauze was placed for urination and head support.

The extraction involved luxation with a Hollenback and removal using mosquito forceps, followed by cleaning the alveolus with a round bur to eliminate root debris. After exodontia, 2g of metallic particles were introduced into 20 alveoli to simulate implant threading. The remaining 20 alveoli served as controls without particle insertion. The 40 total grams after characterizing their size and specific surface area were weighed in 2 grams on a Sartorius 3500 precision balance (Barcelona, Spain). The particles were not washed since they were particles coming from the implantoplasty of the implants and that was the way to be more faithful to the clinical situation. They were introduced into the sockets with a commercially pure titanium spatula.

Absorbable sutures were applied on the following planes: Periosteal (4/0), sub-epidermal (4/0), and skin (2/0). Keeping as near as possible to the edge, simple stitches were inserted. By applying a sterile saline solution, it was possible to maintain the wound in a clean condition. Anti-inflammatory analgesia in the form of buprenorphine 0.05 mg/kg and carprofen 1 mL/12.5 kg was administered.

Post-surgery, the animals were housed individually to prevent aggression and monitored daily for recovery. After 20 days, the animals were euthanized with intra-cardiac sodium pentobarbital, following sedation, and mandibulectomies were per-formed. The samples were fixed in 10% formaldehyde.

Samples were dehydrated in graded ethanol (70%, 80%, 96%, 100%) and embedded in polymethyl methacrylate (PMMA) using Technovit 7200VLC (Zulzer, Germany). Sections were cut longitudinally with a diamond saw (Exact 300-310, Exakt Apparatebau GmbH, Germany) to a final thickness of 30 m and polished with SiC abrasive papers (P400, P800, P1200).

Histological analysis was performed using Masson-Goldner staining. A Zeiss Axio (Axio, USA) imaging system with IMAGE J software (version 1.43, NIH, USA) was used to calculate the percentage of bone-to-implant contact (pBIC). The results were normalized using a modified formula that considered pre-existing bone levels.

Surface topography was analyzed using a JSM 6400 SEM (Jeol, Japan) equipped with EDX microanalysis to determine the chemical composition. The SEM employed a stitching technique, capturing over 750 high-resolution images. Retrodispersive electrons were used to differentiate tissue types.

Statistical analysis

All assays were performed in triplicate, except ALP activity, conducted in quadru-plicate. Results were expressed as mean±standard deviation (SD). Statistical analysis was conducted using MINITAB® (version 18, Minitab Inc.).

Given that homoscedasticity was not confirmed (via Bartlett and Levene's tests), nonparametric tests were applied. The Kruskal-Wallis test was used for multiple com-parisons, while the Mann-Whitney U-test was employed for pairwise comparisons. A significance threshold of p&lt;0.05 was considered.

## Results

Titanium particles characterization

**Figure 1:**
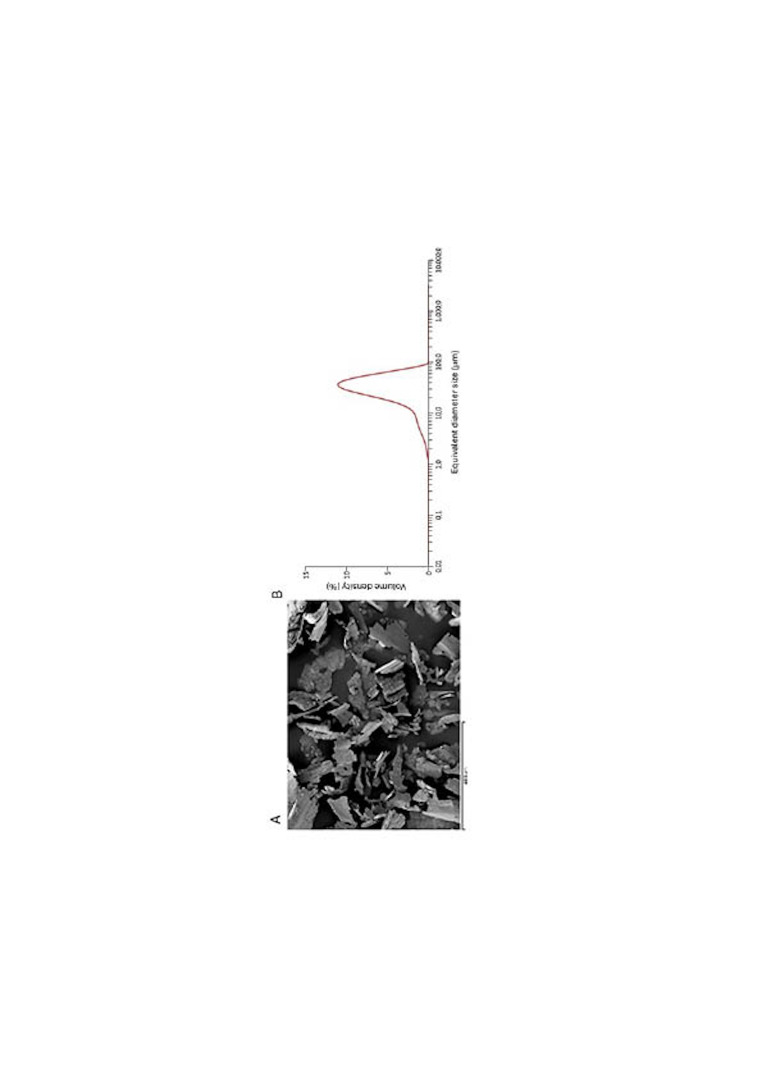
A) Particles released by the IP process observed by SEM. B) Granulometry of the particles released.

Figure 1 Illustrates the morphology of the particles obtained during the IP process. The particles exhibit a predominantly planar morphology, consistent with the characteristics expected from the mechanical machining procedures employed. EDS analyses confirmed the composition of the particles as cap Ti, with no detectable residues from the burs used during the machining process.

1

The particle size analysis revealed a distribution ranging from 1 to 100 micrometers, exhibiting a normal size distribution. The average equivalent diameter was calculated to be approximately 45 micrometers, with the size distribution curve indicating that 90% of the particles fall within the range of 30 to 60 micrometers (Figure 1). Additionally, the specific surface area measurements for the c.p. Ti particles are presented in Table 1.

Table 1

Cell cultures

**Figure 2:**
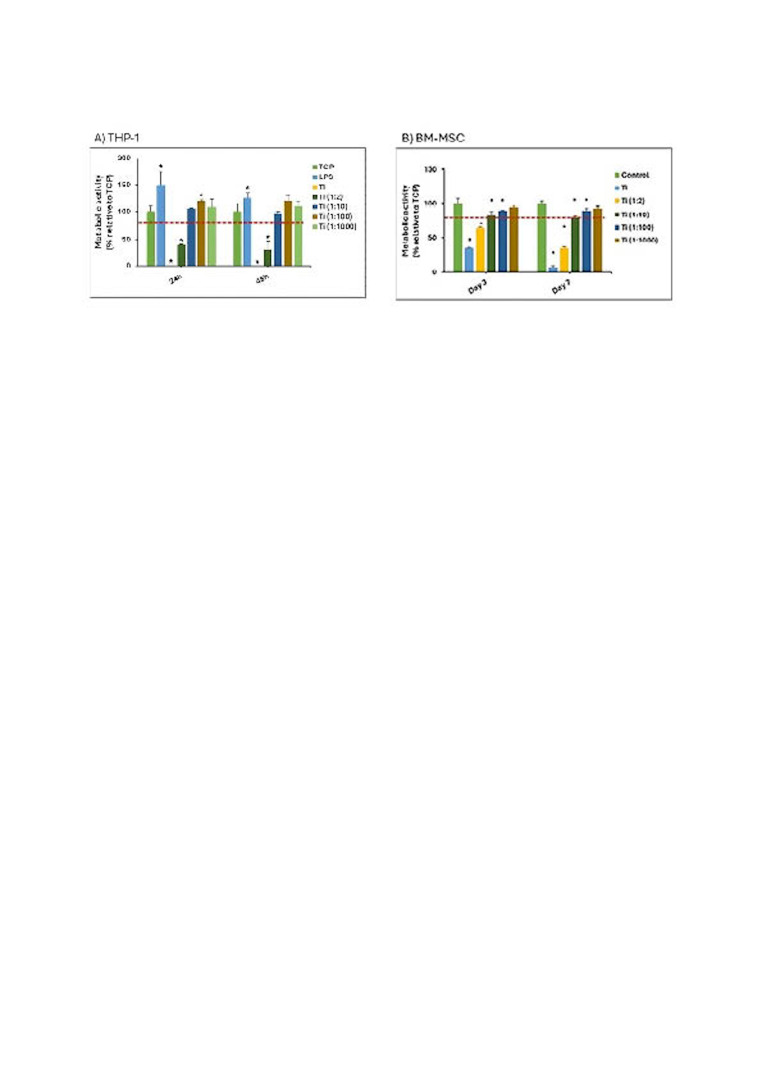
A) Effect of Ti upon THP-1 cell metabolic activity after 24 h and 48 h culture. Metabolic activity results were represented as percentage relative to an unstimulated control (TCP) and compared with the TCP of each day. Values &lt; 80% metabolic activity (red line) which were significantly different (p&lt;0.05) from TCP were considered cytotoxic. Statistically significant differences (p&lt;0.05) are represented with *. LPS: Lipopolysaccharide TCP: Tissue culture plates; Ti: Titanium. B) Effect of Ti particles upon the metabolic activity of human bone marrow-derived mesenchymal stem cells (BM-MSCs) cultured for 3 and 7 days. Metabolic activity results were represented as percentage relative to an unstimulated control (TCP) and compared with the TCP of each day. Values &lt; 80% metabolic activity (red line) which were significantly different (p&lt;0.05) from TCP were considered cytotoxic. Statistically significant differences (p&lt;0.05) are represented with *.

The inflammatory behavior of titanium (Ti) particles was assessed through cytotoxicity tests (Figure 2). Undiluted extracts and 1:2 dilutions exhibited cytotoxic effects on cells at 24 and 48 hours. The 1:100 dilution, identified as non-cytotoxic, was used for subsequent inflammatory response evaluations.

2

**Figure 3:**
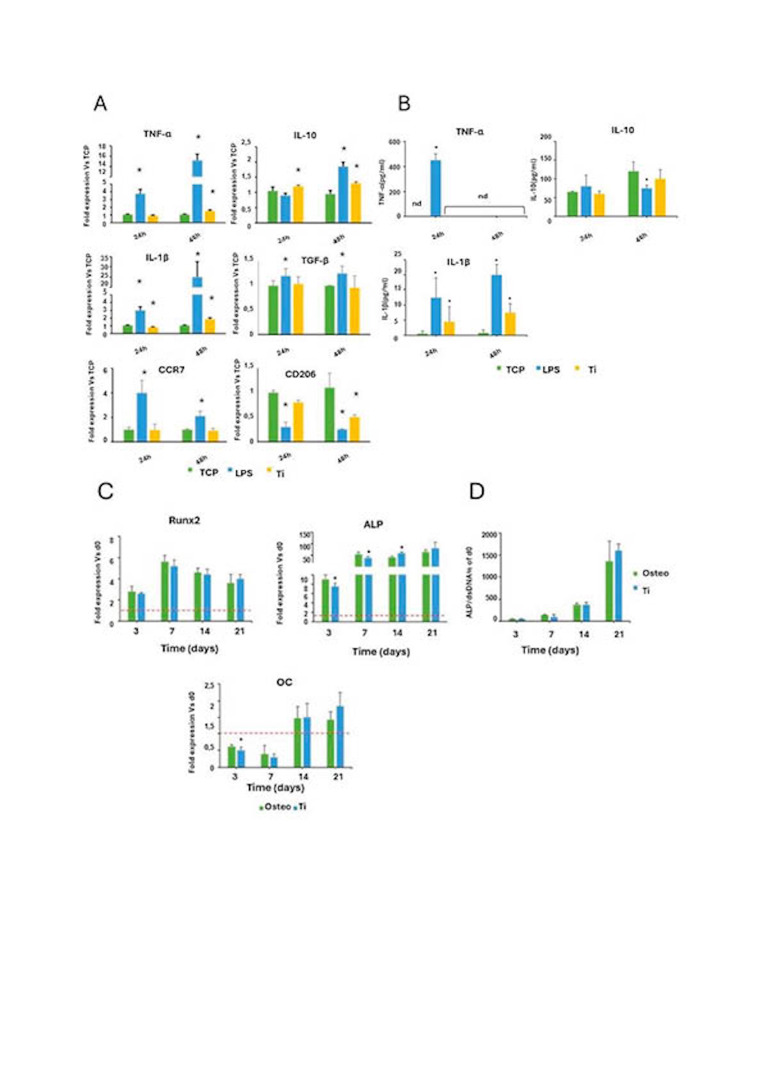
A) Effect of stimulation with Ti extracts upon gene expression of proinflammatory (TNF-α, IL-1β and CCR7) and anti-inflammatory markers (IL-10, TGF-β and CD206) in the THP-1 macrophage cell line. Statistically significant differences (p&lt;0.05) are represented with * when comparison was made versus TCP and with the symbol # when comparison was made versus Ti. B) Effect of stimulation with Ti extracts upon the release of proinflammatory (TNF-α and IL-1β) and anti-inflammatory cytokines (IL-10) in the THP-1 macrophage cell line. Statistically significant differences (p&lt;0.05) are represented with * when comparison was made versus TCP. C) Effect of stimulation with Ti extracts upon gene expression of osteogenic markers Runx2, ALP and OC in cultured human bone marrow-derived mesenchymal stem cells (BM-MSCs). Statistically significant differences (p&lt;0.05) are represented with * when comparison was made versus osteogenic medium (Osteo). D) Effect of stimulation with Ti extracts upon ALP activity in cultured human bone marrow-derived mesenchymal stem cells (BM-MSCs). Statistically significant differences (p&lt;0.05) are represented with * when comparison was made versus TCP.

Lipopolysaccharide (LPS) stimulation significantly increased the expression of pro-inflammatory markers (TNF-, IL-1, CCR7) and decreased the anti-inflammatory marker CD206 (Figure 3). When macrophages were exposed to Ti extracts, TNF- expression increased at 48 hours, while IL-1 exhibited a biphasic response (decreasing at 24 hours and increasing at 48 hours). IL-10 expression also increased, indicating a complex inflammatory response.

Cytokine release analysis revealed that only LPS induced a significant TNF- release at 24 hours, while IL-1 levels were significantly higher in both LPS and Ti extract conditions compared to controls. No significant differences in TNF- release were observed with Ti extracts compared to controls (Figure 3).

3

For osteogenic responses, BM-MSCs exposed to undiluted Ti extracts and 1:2 dilutions exhibited cytotoxic effects at 3 and 7 days (Figure 2B). Non-cytotoxic dilutions (1:10 and 1:100) were used for further analyses. Gene expression showed no significant differences in Runx2 expression, but OC expression was reduced at day 3. ALP expression decreased at days 3, 7, and 14, but increased significantly at day 14 with Ti extracts compared to controls (Figure 3C). ALP activity did not show significant differences compared to osteogenic medium controls (Figure 3).

In vivo test

**Figure 4:**
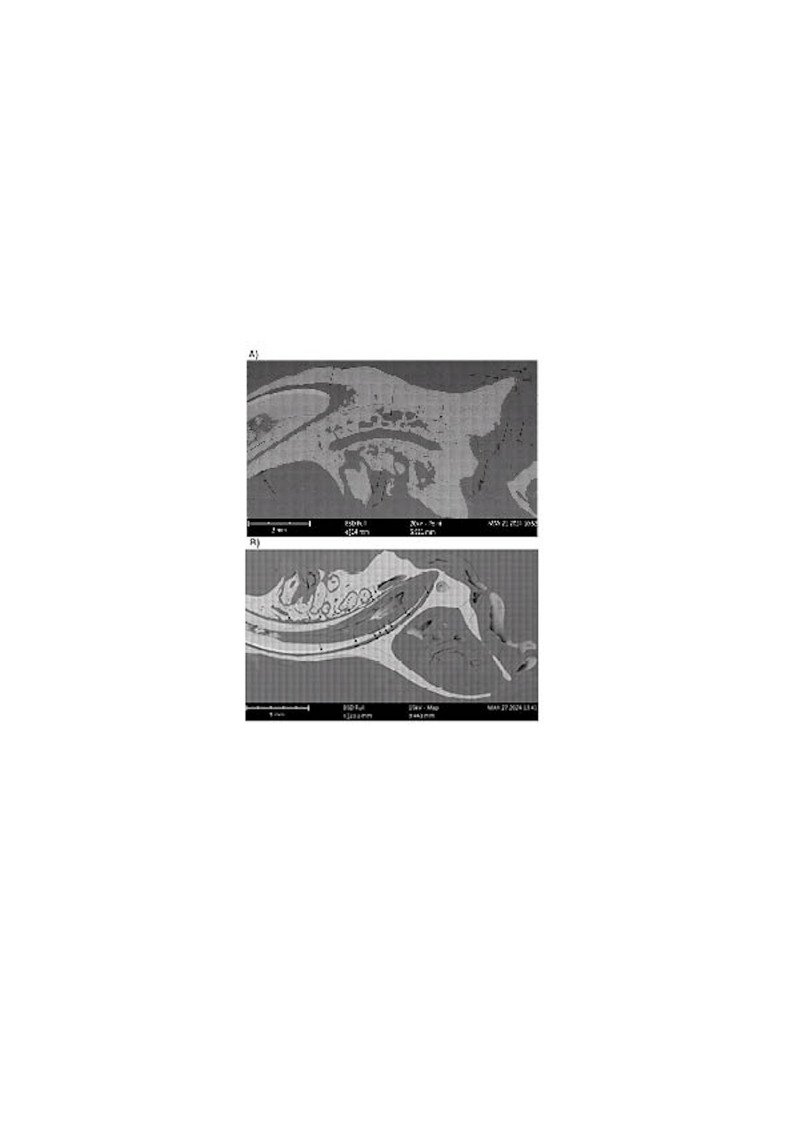
A) Histology observed by SEM. The lighter area correspondsto bone tissue and the darker area is soft tissue. The arrows indicate the position of the particles enveloped in soft tissue. B)Titanium debris encapsulated in soft tissue oriented in the boundary with the bone.

In Wistar rats, inflammatory reactions were observed at sites where titanium (Ti) particles were implanted, with no mortality reported. Histological analyses revealed that the particles were encapsulated within soft tissue and surrounded by a matrix of soft and hard tissues (Figure 4). No evidence of osseointegration was observed. Ti particles occasionally aligned within specific regions, creating boundaries that limited hard tissue formation, suggesting fibrotic encapsulation as the primary immune response (Figure 4).

4

Histological sections exhibited cracks attributed to polishing and vacuum preparation processes. Detached particles left voids matching their original morphology and dimensions. Variations in encapsulated particle sizes were noted, and high-magnification imaging confirmed fibrotic encapsulation of the particles.

Backscattered electron imaging highlighted contrasting tissue densities. Lighter areas corresponded to bone tissue with higher atomic weight, while darker areas represented soft tissue. Encapsulation zones measured approximately 10 micrometers in diameter, confirming the confinement of Ti particles to soft tissue without bone integration.

## Discussion

This study evaluated the inflammatory and osteogenic responses induced by Ti particles generated during the IP process. The immunological characteristics triggered by Ti debris released during IP were analyzed using THP-1 macrophages and BM-MSCs. The findings indicate that c.p. Ti particles can elicit an inflammatory response by promoting the expression and release of proinflammatory genes and cytokines, consistent with previous reports (12 , 13).

Although this study was conducted in accordance with ISO 10993-5 standards for cellular assays, its in vitro design inherently excludes certain clinical variables. Factors such as bacterial contamination or the effects of the oral environment-specifically variations in temperature, pH, and humidity-could exacerbate the inflammatory response (14). Future investigations should address these limitations through in vivo studies, enabling a more comprehensive evaluation of the immune response triggered by Ti particles released during IP and improving the external validity of the findings. Additionally, the mechanical grinding process alters the properties of Ti, increasing its susceptibility to corrosion. This phenomenon, particularly relevant when using remnants of c.p. Ti implants, warrants further investigation to understand its clinical implications (3).

The vast majority of osseointegrated dental implants are manufactured from c.p. Ti, Ti6Al4V alloy, or the more recently introduced Ti-Zr alloy (15). Ti is extensively utilized in dental implantology and orthopedics due to its high biocompatibility, corrosion re-sistance, and favorable mechanical properties (16 , 17). However, concerns have emerged regarding the role of Ti and its metal particles in the development of aseptic osteolysis and peri-implantitis. In this context, the inflammatory and osteogenic responses triggered in vitro by Ti debris released during IP were evaluated.

Ti particulate extracts demonstrated cytotoxicity to THP-1 macrophages and BM-MSCs at concentrations exceeding a 1:10 dilution, with significant reductions in cell viability observed at 24 and 48 hours for THP-1 cells and at 3 and 7 days for BM-MSCs. Proinflammatory responses were evident, marked by increased macrophage polarization toward the M1 phenotype, as indicated by upregulation of TNF-, IL-1, and CCR7, along with reduced expression of the anti-inflammatory marker CD206. These observations align with previous studies, such as Eger et al. (18), which reported that metal particles engulfed by macrophages promote the release of proinflammatory cytokines, including TNF-, IL-6, and IL-1, ultimately leading to osteoclastogenesis via RANKL expression and subsequent bone resorption (19 , 20).

The osteogenic response was also affected by Ti particles. While ALP activity, a key marker of bone mineralization, showed no significant differences compared to the osteogenic medium control, BM-MSC viability decreased, potentially due to apoptosis mediated by tumor suppressor proteins p53 and p73. Gene expression analysis revealed a slight downregulation of Runx2, a critical transcription factor for osteoblast differentiation, and osteocalcin (OC), which is involved in the later stages of bone formation (21 , 22).

Histological analysis demonstrated that Ti particles were encapsulated within soft tissue with no evidence of osseointegration or tissue pigmentation. Unlike the metalosis commonly observed in orthopedic scenarios involving friction-generated high-energy particles, IP-generated Ti particles did not exhibit similar phenomena, as the absence of wear-induced heat prevents the production of black corrosion products (23 , 24). Nevertheless, the deformation caused by IP increased the susceptibility of Ti particles to corrosion, consistent with findings by Toledano-Serrabona et al. (25 , 26) and Lozano et al. (27), which also documented elevated Ti ion release into surrounding tissues.

Several limitations were identified in this study. First, the 30-day observation period was insufficient to evaluate long-term effects. Additionally, the experimental model, which involved filling mandibular defects with Ti particles, does not fully replicate the clinical conditions of IP. Furthermore, particles smaller than 1 µm were not detectable using scanning electron microscopy, leaving their potential systemic effects unexamined. Lastly, variability in IP techniques-such as drill sequence, applied mechanical stress, and the material composition of Ti or its alloys-adds complexity to the interpretation of the results. Future research should focus on strategies to reduce the generation of harmful particles and assess their effects in vivo over extended periods to provide a more thorough understanding of their clinical implications.

## Conclusions

In conclusion, metal particles released during IP showed cytotoxic effects. Proinflammatory markers (TNF-, IL-1) increased over time, while anti-inflammatory marker CD206 decreased. BM-MSCs also exhibited cytotoxicity, but no significant differences in ALP activity were observed compared to the osteogenic control. Histological analysis revealed that Ti particles were encapsulated in soft tissue as part of the immune response, with no evidence of osseointegration or pigmentation.

## Figures and Tables

**Table 1:** Table Specific surface of the Ti particles.

Material	Specific Surface (m2/g)	Correlation Coefficient
Ti	0.4305 ± 0.037	0.9998

1

## Data Availability

The original contributions presented in the study are included in the article, further inquiries can be directed to the corresponding author.
